# PocketMaize: An Android-Smartphone Application for Maize Plant Phenotyping

**DOI:** 10.3389/fpls.2021.770217

**Published:** 2021-11-25

**Authors:** Lingbo Liu, Lejun Yu, Dan Wu, Junli Ye, Hui Feng, Qian Liu, Wanneng Yang

**Affiliations:** ^1^Wuhan National Laboratory for Optoelectronics, Britton Chance Center for Biomedical Photonics, Key Laboratory of Ministry of Education for Biomedical Photonics, Department of Biomedical Engineering, Huazhong University of Science and Technology, Wuhan, China; ^2^School of Biomedical Engineering, Hainan University, Haikou, China; ^3^National Key Laboratory of Crop Genetic Improvement, National Center of Plant Gene Research, Huazhong Agricultural University, Wuhan, China; ^4^Shenzhen Branch, Guangdong Laboratory for Lingnan Modern Agriculture, Genome Analysis Laboratory of the Ministry of Agriculture, Agricultural Genomics Institute at Shenzhen, Chinese Academy of Agricultural Sciences, Shenzhen, China

**Keywords:** smartphone, application, plant phenotyping, deep learning, maize plants

## Abstract

A low-cost portable wild phenotyping system is useful for breeders to obtain detailed phenotypic characterization to identify promising wild species. However, compared with the larger, faster, and more advanced in-laboratory phenotyping systems developed in recent years, the progress for smaller phenotyping systems, which provide fast deployment and potential for wide usage in rural and wild areas, is quite limited. In this study, we developed a portable whole-plant on-device phenotyping smartphone application running on Android that can measure up to 45 traits, including 15 plant traits, 25 leaf traits and 5 stem traits, based on images. To avoid the influence of outdoor environments, we trained a DeepLabV3+ model for segmentation. In addition, an angle calibration algorithm was also designed to reduce the error introduced by the different imaging angles. The average execution time for the analysis of a 20-million-pixel image is within 2,500 ms. The application is a portable on-device fast phenotyping platform providing methods for real-time trait measurement, which will facilitate maize phenotyping in field and benefit crop breeding in future.

## Introduction

Maize (Zea mays L) is one of the essential crops cultivated primarily for food, animal feed, and biofuel, and a more significant amount of maize by weight is produced each year than any other grain (Ritchie and Roser, 2020). Maize plant traits, such as plant architecture, plant biomass, plant projected area, and plant height, are essential factors in the study of maize biology, growth analysis, and yield estimation (Golzarian et al., 2011). Leaves are the primary photosynthetic organs and fundamental importance to maize, acting as transporters, carrying essential materials and energy from the environment, and eliminating waste products (Efroni et al., 2010). Thus, leaf traits, such as leaf area, leaf shape, and leaf number, are also of great significance in maize breeding (Yang et al., 2013). Moreover, the traits of each individual leaf at different heights contribute differently to the final yield prediction (Zhang et al., 2017).

Wild species related to agricultural crops (CWR, crop wild relatives) represent a large pool of genetic diversity, providing new allelic variation for yield improvements, disease resistance, farming practices, and market demands (Dempewolf et al., 2017). The advent of next-generation sequencing technology has resulted in a significant improvement in genomics (Koboldt et al., 2013) and implemented high-throughput genome sequencing for CWR. However, there are substantial gaps in accessible CWR in gene banks, and available evidence indicates that the crop diversity present in farmers’ fields has declined, leading to the rareness or even disappearance of many farmers’ varieties and landraces (FAO, 2010; Pilling et al., 2020). Among the most critical crops across the global food supply, such as wheat, rice, and soybean, maize wild relatives gain the highest priority for further collection to improve their representation in gene banks (Castañeda-Álvarez et al., 2016). When breeders collect CWR resources, a portable device that can provide detailed phenotypic characterization on device in wild conditions is urgently needed.

Over the past few decades, many versatile and high-throughput phenotyping platforms have been developed (Yang et al., 2020). Compared with other phenotyping trait collection methods, image-based phenotyping is noninvasive, scalable, and easy to automate (Das Choudhury et al., 2016). Granier et al. (2006) developed one of the first automated visible-light imaging systems called PHENOPSIS for detecting Arabidopsis responses to water deficit in 2003. Walter applied soil-filled rhizoboxes to make the root visible and established GROWSCREEN for both aboveground and belowground phenotyping in 2007 (Walter et al., 2007). Later, a chlorophyll fluorescence imaging system was attached to the platform, and GROWSCREEN was updated into GROWSCREEN FLUORO, allowing the phenotyping of leaf growth and chlorophyll (Jansen et al., 2009). In the next few years, larger-scale phenotyping platforms in the laboratory, such as Phenoscope (Tisné et al., 2013) and Phenovator (Awlia et al., 2016), were designed for potted plants. These platforms combined the rotating imaging table for multiangle imaging, a high-speed x–y rail system for camera movement (camera to plant) or plant movement (plant to camera), and a dark acclimation chamber for a more stable imaging environment. In general, phenotyping systems in the laboratory are rapidly developing and contain more advanced sensors for additional traits unable to be acquired before. However, indoor phenotyping platforms are costly, time-consuming, immovable, and require skilled engineers for maintenance. To provide phenotyping measurements in the field, a portable, simple-to-operate, and cost-effective phenotyping platform is needed.

Taking advantage of advances in sensors and chip computation power, modern smartphones have become a new solution that combines sensors, platforms, and processing, and a few methodologies for phenotyping with smartphones have been developed (Araus and Kefauver, 2018). The fractional vegetation cover can be estimated from simple calculations with traditional RGB images taken above crop canopies using the smartphone’s own processing capacities (Patrignani and Ochsner, 2015; Chung et al., 2017). PocketPlant3D uses the device accelerator and magnetometer to measure the leaf insertion angle and the leaf angles from the insertion to the tip (Confalonieri et al., 2017). PocketLAI acquires real-time images from below the plant canopy. It uses the smartphone accelerator to obtain the smartphone’s current depression angle and detect sky pixels when the angle between the vertical and the normal to the screen reaches 57° to estimate plant LAI (Orlando et al., 2016). PocketN estimates plant nitrogen content from digital images (Confalonieri et al., 2015). The iPad application “Estimate” takes images of a single expanded leaf and uses standard area diagrams (SADs) to estimate the severity of Cercospora leaf spot (Pethybridge and Nelson, 2018). Some researchers develop applications to acquire images and send them to a server for advanced data processing to transfer machine learning approaches to smartphone applications (Singh et al., 2018). This client-server architecture fills the smartphone computation capacity gap by transmitting image data to an in-house server for advanced image processing to detect Cercospora leaf spots on sugar beet (Hallau et al., 2018). A cloud-based system that can send the images taken from the greenhouse to the cloud is developed for water stress prediction using window-based support vector regression (multimodal SW-SVR) (Kaneda et al., 2017). These applications revealed the potential of mobile devices in plant phenotyping.

In previous work, our phenotyping team developed a high-throughput indoor phenotyping facility called HRPF to extract rice phenotypic traits (Yang et al., 2014), and more novel imaging techniques were renewed and applied in more crops, such as multiangle RGB imaging for 3D reconstruction of wheat plants (Fang et al., 2016), hyperspectral imaging for rice plant biomass (Feng et al., 2013) and rice leaf chlorophyll (Feng et al., 2013), and micro-CT for rice tiller traits (Wu et al., 2019). In the present work, we developed a portable on-device phenotyping system running on Android to nondestructively extract 15 plant traits, 25 leaf traits and 5 stem traits with high efficiency (up to 3 s per plant), which provides a real-time quantitative maize trait analysis for breeders.

## Materials and Methods

### Material and Experimental Design

The study area was located at Huazhong Agricultural University, Wuhan, Hubei Province, China (30.5N, 114.3E) at an average elevation of 16 m. The maize variety of JinZhongYu (YT0213/YT0235) was sown and germinated during the summer of 2015. Ninety maize plants were planted in a plastic pot and in the field. The pots were 23.5 cm in diameter and 35 cm in height with approximately 6 l of experimental soil (pH 5.45, total nitrogen 0.241 g kg^–1^, total potassium 7.20 g kg^–1^, total phosphorus 0.74 g kg^–1^, alkali-hydrolysable nitrogen 144.06 mg kg^–1^, available potassium 188.64 mg kg^–1^, available phosphorus 16.81 mg kg^–1^, organic matter 46.55 g kg^–1^).

The measurement started 30 days after sprouting. Every 3 days, nine plants in the pots and nine plants in the field were randomly picked and photographed outdoors via an application (PocketMaize) running on an ANDROID smartphone (MEIZU, MX4). A portable black backdrop is placed behind the plant as background, and a marker object is placed next to the plant to calculate the resolution between image pixels and the real world. Four images with pitch angles of approximately 0° (front view), 10°, 15°, and 20° were taken for each plant with arbitrary distance from camera to the plant and imaging height. Images were stored in JPG format with a resolution of 3,936 × 5,248 pixels. The app stores the pictures and records the current spatial angle, time, and date when a picture is taken. Other necessary pieces of information, such as plant ID, can also be manually input.

After imaging, plant height was manually measured with a ruler vertically placed against the plastic pot’s edge or the field ground on top of the soil surface. The shoot part of the maize plant was then cropped down for destructive measurements. The fresh leaf biomass and stem biomass were estimated separately. The individual leaves of each plant were cut, and the leaf area was measured using high-throughput leaf scoring (HLS) (Yang et al., 2015). Then, the plants were sealed and oven-dried for further dry-weight determination.

### The Image Process Procedure

**FIGURE 1:**
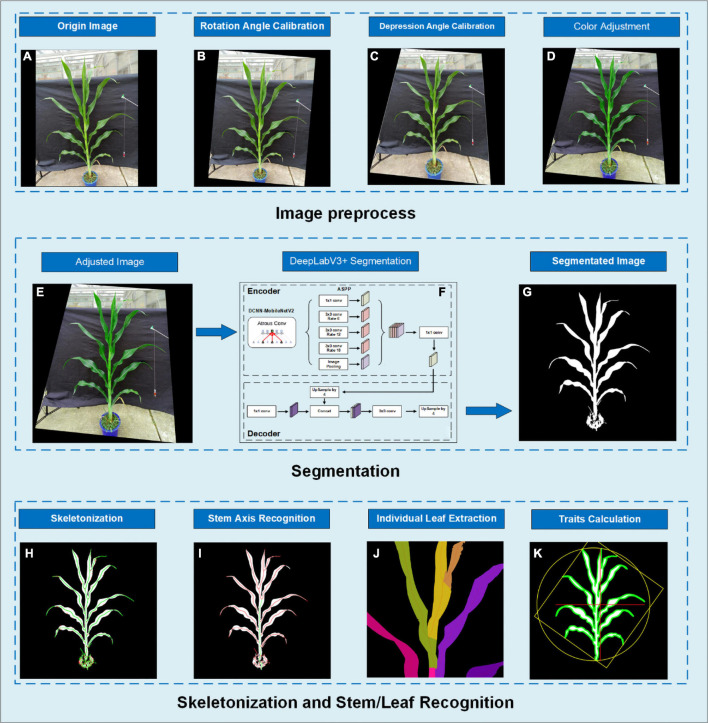
The image analysis pipeline showing **(A)** original image; **(B)** rotated image to calibrate rotation angle; **(C)** depression angle calibration using perspective transform; **(D)** color adjustment; **(E)** the resulting image of preprocessing; **(F)** segmentation using DeepLabV3+; **(G)** segmentation result image; **(H)** skeletonization using our distance transform-based algorithm; **(I)** stem axis recognition by finding the overlaid route; **(J)** pixel extraction for each individual leaf; **(K)** result image.

In this study, we developed an application running on Android smartphones (called PocketMaize) for image acquisition, image processing, and plant traits extraction combined (Figure 1). Image processing’s key steps include image calibration, angle calibration, image segmentation, skeletonization, stem and leaf extraction, and phenotypic traits calculation.

The first step, camera distortion calibration using an OpenCV calibration function (Zhang, 2000), is optional. A black and white calibration pattern pasted on a plastic plate was used to obtain 20–25 images. Furthermore, the imaging angles between each image should have apparent differences to ensure accuracy.

**FIGURE 2:**
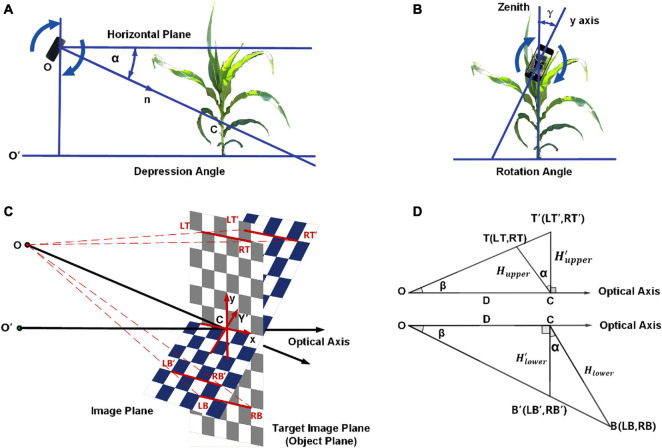
The calibration of rotation angle and depression angle showing **(A)** explanation of depression angle; **(B)** explanation of rotation angle; **(C)** schematic diagram for depression angle; **(D)** longitudinal section for depression angle.

The second step is to calibrate the rotation angle and depression angle. As shown in Figures 2A,B, the depression angle **α** is the angle between the normal n to the plane on which the device’s screen lays and the horizontal plane, while the rotation angle **γ** is the angle between the y-axis of the screen and the zenith.

An ideal image for trait extraction should be perpendicular to the ground and have the same object-pixel resolution for the whole image. One of the best options is orthographic projection imaging. For most of the other image-based phenotyping systems, camera lens distortion calibration is sufficient because in these systems, the cameras are fixed to obtain a stable imaging angle. However, in our application, the position and direction of the camera are continuously changing, making it essential to calibrate ordinary images with different rotation and depression angles to an approximate orthographic projection image by image transformation.

Gravity sensors in the smartphone provide live data of the rotation angle and depression angle, and these two angles are calibrated separately. Figure 1A shows an original image obtained from camera calibration whose rotation angle and depression angle need to be calibrated. Usually, the perpendicularity is satisfied by simply rotating the image clockwise or anticlockwise using the rotation angle obtained from the accelerometer to match the gravity direction while a perspective transform was applied to adjust the depression angle. Figures 1B,C display the results of the rotation angle calibration and depression angle calibration, respectively.

The perspective transform is used in depression angle calibration, which is a nonlinear geometric transformation that can change an image from one viewpoint to another viewpoint or, in other words, change the position of the image plane. It is widely used in image processing, including length calculation, marking recognition in images (Liu et al., 2012) and vision guidance for vehicles (Torii, 2000). Perspective transform can transform the ordinary images taken in this experiment with different imaging angles to an approximate orthographic projection image. It needs the coordinates of four sets of points, four given points on the original image plane and their corresponding points on the target image plane, to calculate the perspective transform matrix. Placing four markers on to the background can reduce obstacles in image processing. The markers might be obscured by leaves, and it is troublesome to determine the relative positions in the wild. Therefore, a camera calibration method without markers was developed.

Examine a 2W × 2H rectangle with four symmetric corner points *LT, RT, LB, RB* on the target image plane (object plane) and their corresponding points *LT*′, *RT*′, *LB*′, *RB*′ on the origin image plane. Figure 2C shows the position of these eight points, and point C is the origin point of the coordinate system on both the object plane and image plane. The coordinates of these eight points are *LT*(−*W*,*H*), *RT*(*W*,*H*), *LB*(−*W*,−*H*), and *RB*(*W*,*H*) on the target image plane and $LT^{{}^{\prime }}\left(-W_{upper}^{{}^{\prime }},H_{upper}^{{}^{\prime }}\right)$, $RT^{{}^{\prime }}\left(W_{upper}^{{}^{\prime }},H_{upper}^{{}^{\prime }}\right)$, $LB^{{}^{\prime }}\left(-W_{lower}^{{}^{\prime }},H_{lower}^{{}^{\prime }}\right)$, and $RB^{{}^{\prime }}\left(W_{lower}^{{}^{\prime }},H_{lower}^{{}^{\prime }}\right)$ on the origin image plane. Figure 2D is the longitudinal section at the center, while *T* and *T*′ are the center of *LT, RT* and *LT*′, *RT*′ and *B* and *B*′ are the center of *LB, RB* and *LB*′, *RB*′. First, for the upper part of the image, let’s mark


(1) $$\begin{array}{c} \{\begin{array}{c} L_{upper}=\bar{OT}\\ L_{upper}^{{}^{\prime }}=\bar{OT^{\prime }} \end{array} \end{array}$$

Then, we have


(2) $$\begin{array}{c} \frac{W_{upper}^{{}^{\prime }}}{W}=\frac{L_{upper}^{{}^{\prime }}}{L_{upper}} \end{array}$$

Let β be the actual viewing angle of the point and *D* be the distance between the camera and the plant; then, we have:


(3) $$\begin{array}{c} H_{upper}^{{}^{\prime }}=D\tan\beta \end{array}$$

(4) $$\begin{array}{c} H_{max}^{{}^{\prime }}=D\tan\beta_{max} \end{array}$$

(5) $$\begin{array}{c} \beta =\tan^{-1}\left(\frac{H^{{}^{\prime }}\tan\beta_{max}}{H_{max}^{{}^{\prime }}}\right) \end{array}$$

where β_*max*_ is the half vertical field of view (VFOV) of the camera and $H_{max}^{{}^{\prime }}$ is the half y resolution of the camera.

The trigonometric relationship in the upper half of the image can be described as follows:


(6) $$\begin{array}{c} D=L_{upper}^{{}^{\prime }}\cos\beta \end{array}$$

(7) $$\begin{array}{c} \frac{H_{upper}}{\sin\beta }=\frac{D}{\sin\left[\pi -\beta -\left(\frac{\pi }{2}-\alpha \right)\right]} \end{array}$$

(8) $$\begin{array}{c} \frac{L_{upper}}{\sin\left(\frac{\pi }{2}-\alpha \right)}=\frac{D}{\sin\left[\pi -\beta -\left(\frac{\pi }{2}-\alpha \right)\right]} \end{array}$$

At last, we have


(9) $$\begin{array}{c} L_{upper}^{{}^{\prime }}=\frac{\cos\left(\alpha -\beta \right)}{\cos\alpha \cos\beta }L_{upper} \end{array}$$

and


(10) $$\begin{array}{c} H_{upper}^{{}^{\prime }}=\frac{\cos\left(\alpha -\beta \right)}{\cos\beta }H_{upper} \end{array}$$

Similarly, for the lower half of the image, we have


(11) $$\begin{array}{c} \frac{H_{lower}}{\sin\left[\pi -\alpha -\left(\frac{\pi }{2}-\alpha -\beta \right)\right]}=\frac{H_{lower}^{{}^{\prime }}}{\sin\left(\frac{\pi }{2}-\alpha -\beta \right)} \end{array}$$

(12) $$\begin{array}{c} L_{lower}^{{}^{\prime }}=\frac{H_{lower}^{{}^{\prime }}}{\sin\beta } \end{array}$$

(13) $$\begin{array}{c} \frac{L_{lower}}{\sin\left(\frac{\pi }{2}+\alpha \right)}=\frac{H_{lower}}{\sin\beta } \end{array}$$

Hence, the final proportion is given by


(14) $$\begin{array}{c} H_{lower}^{{}^{\prime }}=\frac{\cos\left(\alpha +\beta \right)}{\cos\beta }H_{lower} \end{array}$$

(15) $$\begin{array}{c} L_{lower}^{{}^{\prime }}=\frac{\cos\left(\alpha +\beta \right)}{\cos\alpha \cos\beta }L_{lower} \end{array}$$

From formulas (9), (10), (14), and (15), for any given point on the original image plane, we can calculate the coordinates of the corresponding points on the target image plane and vice versa. Then, we can calculate the eight points needed to transform the ordinary image to an approximate orthographic projection image.

The color enhancement in this manuscript aims to standardize the image color according to the main color of the target object and enlarge the color difference between the plant part and background. The purpose of color enhancement here is to improve the segmentation result of DeepLabV3+ under different surrounding weather and illumination conditions.

### Use DeeplabV3+ With the MobileNet Backbone for Segmentation

Segmentation of the plant image is the critical step for the image process. We introduced the DeepLabV3+ model with a MobileNet backbone to obtain segmented results of images with different lightness conditions and backgrounds. DeepLabV3+ is a convolutional neural network model designed for pixel-based semantic image segmentation that has three improved versions (Chen et al., 2018).

Convolutional neural networks use several layers of filters convolved with the input data to greatly reduce the dimension of input data and extract features of the image. These features from each layer are combined into feature maps that can then be used to make the output prediction. Compared with other convolutional neural networks, DeepLabV1 (Chen et al., 2016) introduced a dilated convolution to increase the receptive field to regain the data lost in the pooling layer and used the conditional random field (CRF) to improve boundary recognition. DeepLabV2 (Chen et al., 2017) established the model with atrous spatial pyramid pooling (ASPP) to handle images of similar objects with different scales. DeepLabV3 (Chen et al., 2018) adds a batch normalization layer into the ASPP, and DeepLabV3+ uses a simple decoder module to further upgrade boundary recognition. The DeepLabV3 Plus model is a deep convolutional neural network with atrous convolution that can increase the receptive field without increasing the number of parameters or reducing the dimension of space.

**FIGURE 3:**
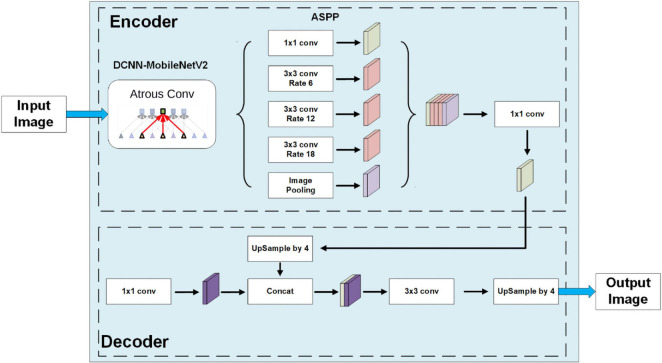
The model structure of DeepLabV3+ with the MobuleNetV2 backbone.

Several kinds of backbones can be used in DeepLab, including ResNet (He et al., 2015), Xception (Chollet, 2017) and MobileNet (Howard et al., 2017). All these models have good performance in maize segmentation, especially Xception. However, to transfer the model to mobile devices, we decided to train our DeepLab model with the most lightweighted MobileNet. The structure of the whole DeepLabV3+ model with the MobileNetV2 backbone is shown in Figure 3. These modules are implemented in TensorFlow (Abadi et al., 2016).

Our own dataset included 720 images in the training set and 80 images in the validation set. A horizontal flip is applied to each image to produce a final training set of 1,440 training and 190 validation images. The transfer training was started with an initialized model pre-trained on the VOC 2012 dataset. The loss weight of the loss function is modified according to the total pixel size of background and the plant. The logit layer and the last layer are excluded to train on our own dataset.

### Thinning Algorithm

**FIGURE 4:**
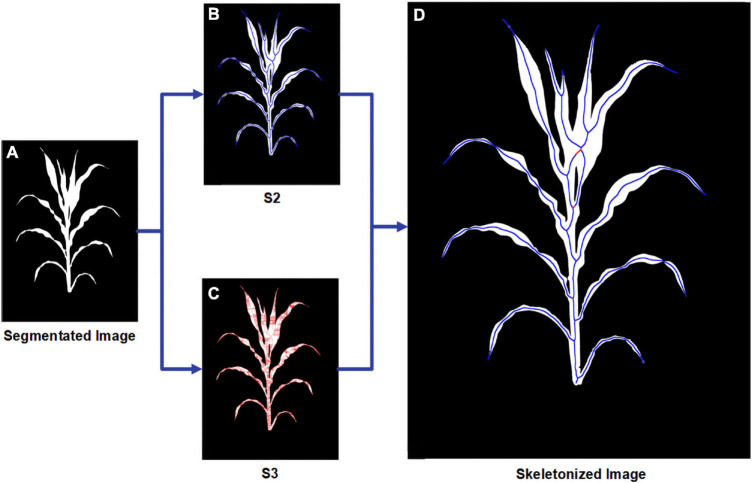
Schematic workflow for the skeletonization procedure showing **(A)** origin segmented image; **(B)** all points in *S*_*2*_; **(C)** all points in *S*_*3*_; **(D)** final result for skeletonization with the points from *S*_*2*_ marked in blue and the points from *S*_*3*_ marked in red.

The media axis of the segmented image is essential for stem and leaf recognition and the calculation of traits such as stem height and leaf length. Thinning/peeling-based methods such as Zhang’s thinning algorithm (Zhang and Suen, 1984) will produce numerous spurs and are time-consuming, and Voronoi diagram-based methods will have difficulty deciding whether a skeletal branch should be pruned. Since the plant’s binary images in this study are relatively large (originally up to 20 million pixels and will become even larger after angle calibration in this manuscript and might be larger for more advanced smartphones) and the boundary of the plant is usually very complicated and meandering, a proper way is to use distance transform-based methods. We developed a two-step skeletonization algorithm (Figure 4) based on the distance transform algorithm (Felzenszwalb and Huttenlocher, 2004). First, a distance transform algorithm was applied to the binary image. Define *S*_*t*_ to be the point set of the target skeleton we needed and *S*_*n*_ to be the point set containing all the points whose value in the distance transformed image is larger than at least n points in its eight neighbors. Figure 4A displays the original segmented image, and Figures 4B,C show the points in *S*_*2*_ and *S*_*3*_. We have approximately *S*_2_⊆*S*_*t*_⊆*S*_3_. In the second step, we designed a path finding algorithm to find a way to connect the points in *S*_*2*_ with the points in *S*_*3*_. Figure 4D is the result of our algorithm with the origin points in *S*_*2*_ marks in blue and the connected pixels from *S*_3_ marks in red.

### Stem and Leaf Extraction

**FIGURE 5:**
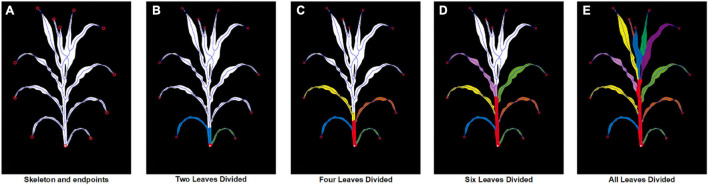
The procedure of leaf pixel segmentation showing **(A)** the original segmented image and skeletonized image; **(B–E)** the segmentation result of two, four, six and all leaves with each leaf marked in different colors and the stem marked in red.

The stem was extracted by finding the shared route connecting the upper part and the plant root, and Figure 5 presents the practical steps. First, all the endpoints of the skeleton image are detected. Figure 5A is the image of the mid axis and all the endpoints. Then, the shortest routes between the lowest endpoint and each of the other endpoints are traced and summed. Figures 5B–E shows this tracing procedure from lower leaves to higher leaves, where each individual leaf is marked in different colors and the overlaid route is marked in red.

Leaf apexes were located at the endpoints of the skeleton image. The leaf direction can be traced by finding the shortest route between leaf apexes and plant stems along the skeleton. Figure 5E displays the segmented plant stem and individual leaves painted in different colors. The leaf insertion angle and the leaf angles from the insertion to the tip can be directly measured from the leaf mid axis. With the stem area removed, the leaves in the lower half were naturally separated.

### Traits Extraction

Finally, from the segmented images and skeletonized images, we calculated 45 traits (Table 1), which included 15 plant traits, 25 leaf traits and 5 stem traits. We can also extract each individual leaf and analyze the difference between leaves at the upper part of the plant and the lower part.

**TABLE 1:** Trait classification and abbreviation.

Trait classification	Trait	Trait abbreviation
Plant traits	Maximum plant height in side view	MPH
	Vertical plant height in side view	VPH
	Plant width in side view	PW
	Total projected area	TPA
	Green projected area/total projected area in side view	GPAR
	Total projected area/bounding rectangle area ratio in side view	TBR
	Plant perimeter in side view	PP
	Perimeter/projected area ratio in side view	PAR
	Plant compactness in side view	PC
	Fractal dimension in side view	FD
	Height to width ratio of minimum circumscribed box in side view	HWR
	The area of convex hull	ACH
	The perimeter of convex hull	PCH
	Plant area/convex hull area	PCHAR
	Total dry weight	TDW
Leaf traits	Total leaf dry weight	LDW
	Total leaf area	TLA
	Total leaf projection area	TLPA
	Total leaf length per plant	TLL
	Leaf number per plant	LN
	Standard deviation of straightened leaf length per plant	SDSLL
	Average distance between the leaf tip and node per plant	LNL
	Standard deviation of the distance between the leaf tip and node per plant	SDLNL
	Average leaf curvature per plant	LC
	Standard deviation of leaf curvature per plant	SDLC
	Average leaf tangency angle per plant	LTA
	Standard deviation of leaf tangency angle per plant	SDLTA
	Average leaf straight angle per plant	LSA
	Standard deviation of leaf straight angle per plant	SDLSA
	Average straightened leaf length in lower half of plant	SLL_below
	Average distance between the leaf tip and node in lower half of plant	LNL_below
	Average leaf curvature in lower half of plant	LC_below
	Average of leaf tangency angle in lower half of plant	LTA_below
	Average of leaf straight angle in lower half of plant	LSA_below
	Average straightened leaf length in upper half of plant	SLL_above
	Average distance between the leaf tip and node in upper half of plant	LNL_above
	Average leaf curvature in upper half of plant	LC_above
	Average of leaf tangency angle in upper half of plant	LTA_above
	Average of leaf straight angle in upper half of plant	LSA_above
	Total leaf dry weight	TLDW
Stem traits	Stem height	SH
	Stem projection area	SPA
	Average stem width	SW
	Stem volume	SV
	Stem dry weight	SDW

## Results

### Development of a Smartphone Application: PocketMaize

In this study, equipped with an Android smartphone (Meizu MX4, MediaTek6595, CPU A17 2.2 GHz × 4 + A7 1.7 GHz × 4, GPU PowerVR G6200), the application was developed with two sensors: an RGB camera and a 3-axis accelerometer. In the image taking mode, an indicator displaying the current depression angle and rotation angle allows users to adjust phone orientation to obtain the appropriate angles. Images were stored in JPG format with a resolution of 3,936 × 5,248 pixels. The camera was autofocused; ISO, shutter speed, and light balance were autofixed. Other necessary information, such as time, date, and plant ID, could also be manually input.

**FIGURE 6:**
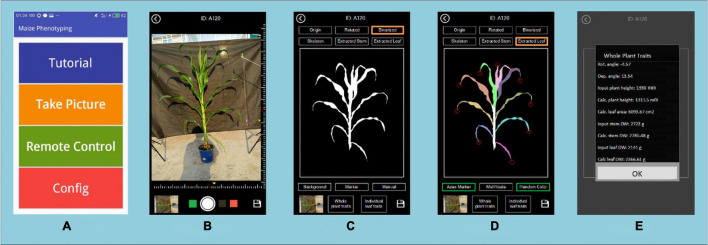
The user interface of PocketMaize showing **(A)** menu; **(B)** image capturing page; **(C)** segmentation result page; **(D)** leaf extraction result page; **(E)** traits extraction result page.

Image processing, processed images and extracted traits can be displayed and saved on the device. The final traits of the maize are stored in a CSV file. Figure 6 shows the user interface of the application, which includes the main menu (A), the image taking page (B), the result of segmentation and stem and leaf recognition (C,D), and the traits displaying page (E).

### Performance Evaluation of DeepLabV3+ Segmentation

**FIGURE 7:**
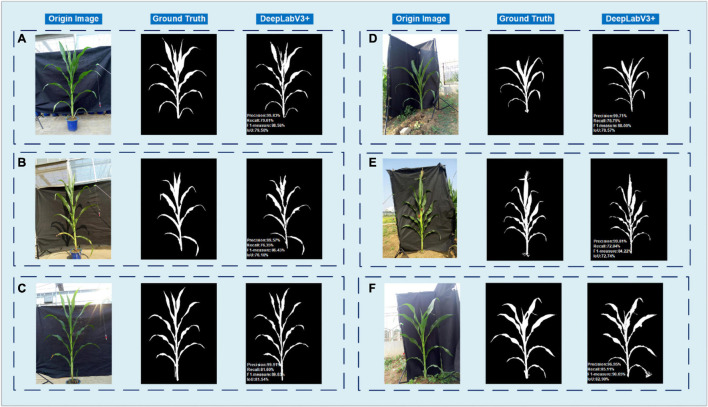
Comparison of manual ground truth and the segmentation result of DeepLabV3+. The left three columns **(A–C)** are the results of three potted samples, and the right three columns **(D–F)** are the results of three in-field samples. The first row **(A,D)** shows samples taken in sunny mornings with even illumination. The second row **(B,E)** shows two samples taken at dawn when the images have a heavy yellow color deviation. The last row **(C,F)** is taken at midday with high brightness.

In this study, after 1,440 images of maize were used to train the DeepLabV3+ model, another 190 images, including 95 images of potted samples and 95 in-field samples, were selected to test the DeepLabV3+ model. To evaluate the performance, four indicators, including precision, recall, F1-measure and IoU, are adopted. Figure 7 shows the results of six samples under different conditions. The left three columns (A–C) are the results of three potted samples, and the right three columns (D–F) are the results of three in-field samples. The first row (A,D) shows samples taken in sunny mornings with even illumination. The second row (B,E) shows two samples taken at dawn when the images have a heavy yellow color deviation. The last row (C,F) is taken at midday with high brightness. In general, the DeepLabV3+ model works well in different color temperatures, different light intensities and mild wind or mild rainy days. However, a sun halo might influence the segmentation result. Although heavy wind will not affect the segmentation stage, it will decrease the accuracy in later stem and leaf recognition stage since the structure of the plant may greatly change.

For the DeepLabV3+ model, the mean values of the Precision, Recall, F1-measure and IoU are 97.31, 74.85, 86.10, and 79.91%, respectively.

### Accuracy Evaluation of Plant Height Measurement

**FIGURE 8:**
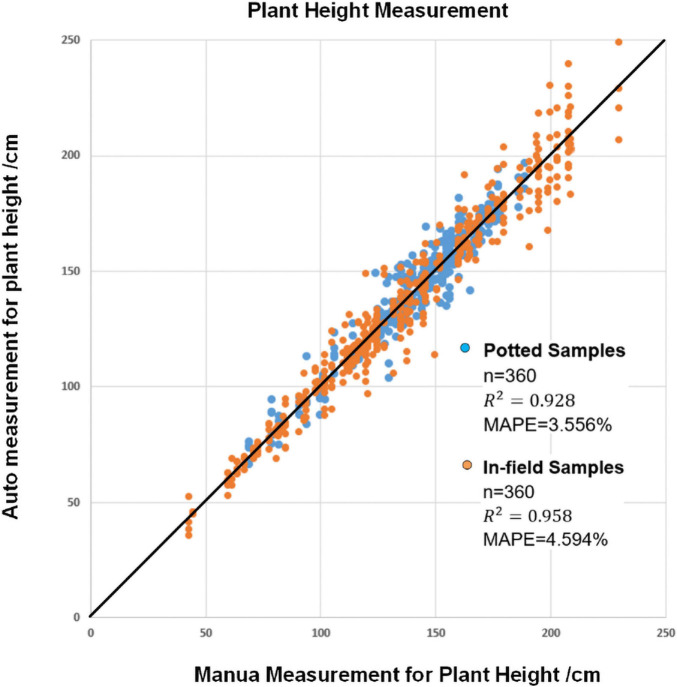
The result of automatic plant height measurement versus manual plant height measurement.

Plant height is the vertical distance from the bottom of the stem at soil surface to the top position of the while plant. To evaluate the measurement accuracy of plant height, all the plants were manually measured. The plant height was measured after the images were captured, and automatic plant height measurement was used to calculate the actual distance between the bottom position of the stem and the top of the whole plant. Figure 8 shows the plant height results of manual observation versus automatic observation in all four depression degrees for all plants. The MAPE values were 3.556% for potted samples and 4.594% for field samples, and the *R*^2^ coefficients were 0.928 and 0.958, respectively. The results show that smartphone applications can correctly detect stems and have good potential for accurate measurement.

### Accuracy Evaluation of Leaf Area and Leaf Dry Weight

**FIGURE 9:**
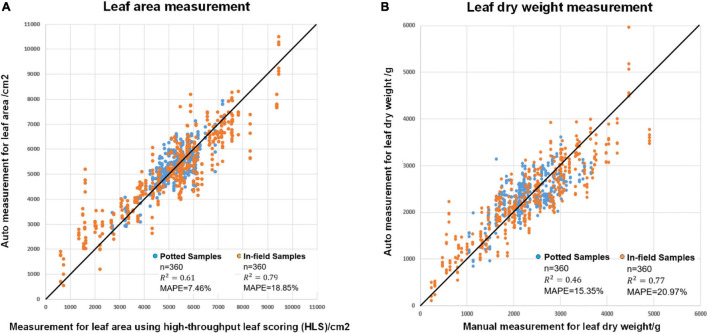
The modeling result of **(A)** automatic leaf area measurement versus measurement using high-throughput leaf scoring (HLS) and **(B)** automatic leaf dry height measurement versus manual leaf dry height measurement.

Figure 9 shows the results of leaf area estimation (A) and leaf dry weight estimation (B). The MAPE values were 7.46% for potted leaf area, 18.85% for in-field leaf area, 15.35% for potted leaf dry weight and 20.97% for in-field leaf dry weight estimation. The squares of the correlation coefficients (*R*^2^) were 0.61, 0.79, 0.46, and 0.77. The detailed model summaries for stepwise regression analysis for leaf area estimation and leaf dry weight estimation are shown in Supplementary Tables 1, 2.

### Accuracy Evaluation of Stem Dry Weight

**FIGURE 10:**
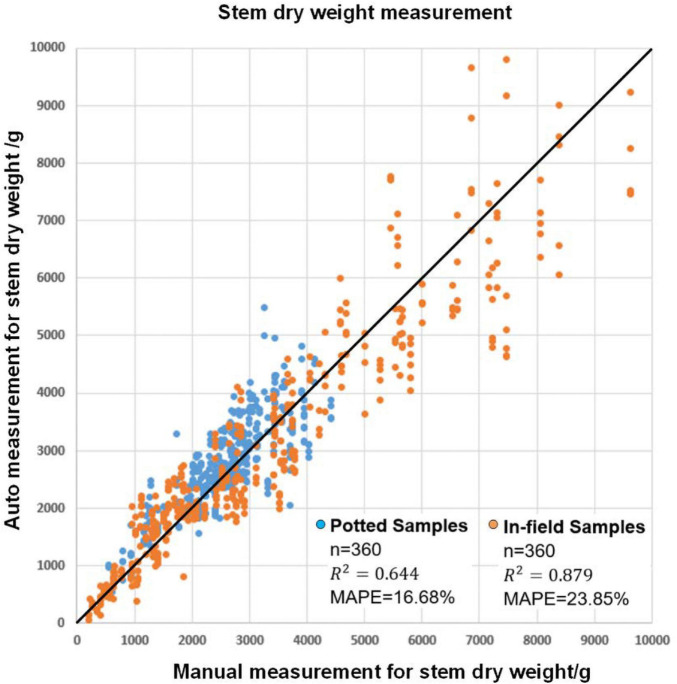
The modeling result for automatic stem dry weight measurement and manual stem dry weight measurement.

Since maize stems and maize leaves have a significant difference in organ structure and density, it is natural to evaluate the stem dry weight and leaf dry weight separately. In particular, the plant stem can be approximately seen as a cylinder, so the plant stem’s dry weight can be estimated with the volume of a cylinder fitted to the stem together with other traits such as stem projected area, stem height and average stem width. Figure 10 shows the result of stem biomass measurement. The MAPE values were 16.68 and 23.85% for potted and field samples, respectively. The squares of the correlation coefficients (*R*^2^) were 0.64 and 0.88, respectively. The detailed model summaries for stepwise regression analysis for leaf area estimation and leaf dry weight estimation are shown in Supplementary Table 3.

## Discussion

### Comparison of Trait Extraction With/Without Depression Angle Calibration

**FIGURE 11:**
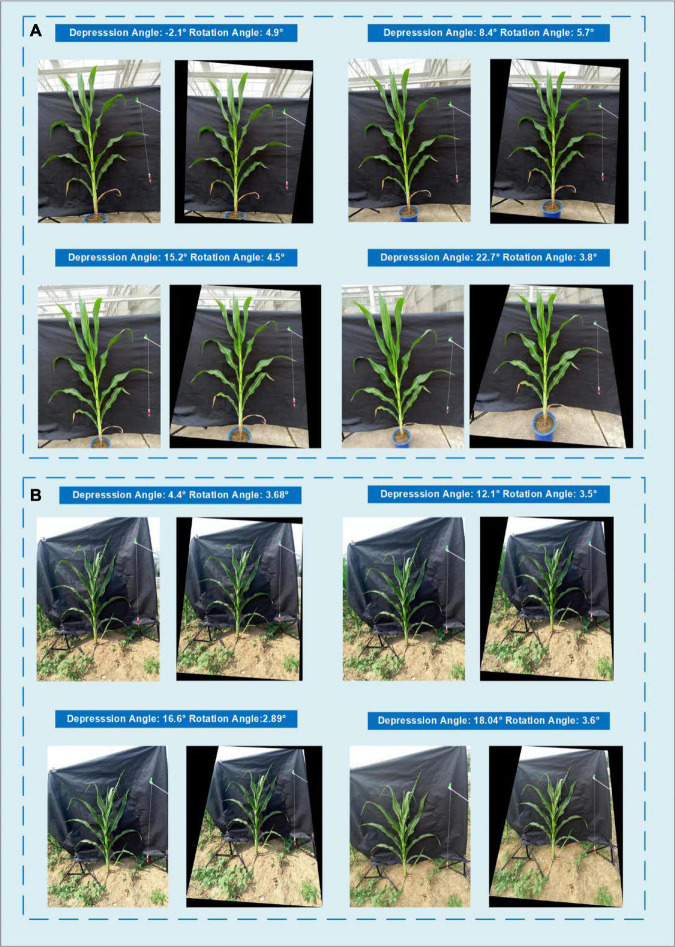
Comparison of the images taken with different depression angles and their angle calibrated results showing **(A)** a potted sample and **(B)** an in-field sample.

Since the difference in the depression angle can greatly change the original image, a depression angle calibration is essential before advanced image processing. Figure 11 shows an example of eight images of one potted sample and one in-field sample with different depression angles and rotation angles. The actual depression angles/rotation angles are −2.1°/4.9°, 8.4°/5.7°, 15.2°/4.5°, and 22.7°/3.8° for the potted sample shown in A and 4.4°/3.68°, 12.1°/3.5°, 16.6°/2.89°, and 18.4°/3.6° for the field sample shown in B. The calibrated images show that our angle calibration algorithm can vastly reduce the influence caused by different rotation and depression angles and transform the plant to an approximate front view.

**FIGURE 12:**
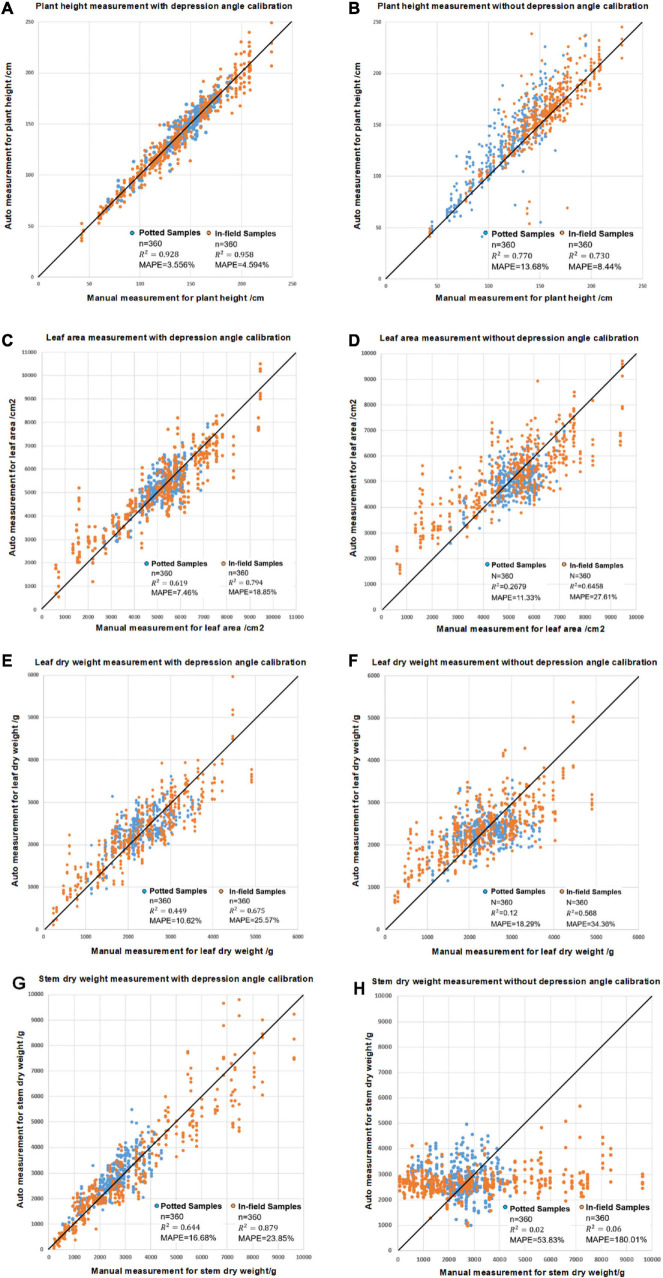
The comparison of the modeling results for leaf area, leaf dry weight and stem dry weight measurements with and without angle calibration showing **(A)** plant height measurements with depression angle calibration, **(B)** plant height measurements without depression angle calibration, **(C)** leaf area measurements with calibration, **(D)** leaf area measurements without calibration, **(E)** leaf dry weight measurements with calibration, **(F)** leaf dry weight measurements without calibration, **(G)** stem dry weight measurements with calibration and **(H)** stem dry weight measurements without calibration.

Figure 12 shows the plant height, leaf area, leaf dry biomass and stem dry biomass with and without depression angle calibration. The results indicate that for plant height, leaf area and leaf dry weight, the result is still meaningful without depression calibration, with *R*^2^ values up to 0.73, 0.65, and 0.57 for in-field samples (Figures 12B,D,F). However, a depression angle calibration can increase the measurement accuracy as *R*^2^ increases to 0.99, 0.79, and 0.68 (Figures 12A,C,E). Stem dry weight can only be measured with depression angle calibration.

### Comparison of Four Skeletonization Methods

**FIGURE 13:**
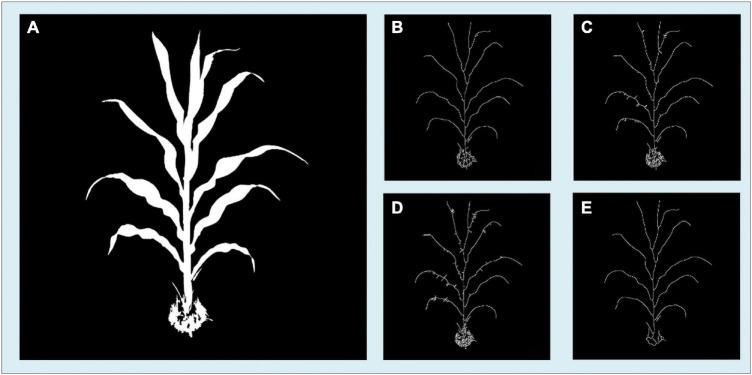
Comparison of the skeletonization results of the four methods showing **(A)** the segmented image. **(B)** Result image of our algorithm. **(C)** Results image of Zhang’s thinning algorithm with branch pruning. **(D)** Result image of Scikit-image’s skeletonization. **(E)** Results image of heat equation-based skeletonization.

The skeleton algorithm we developed is based on the distance transform algorithm. Several existing skeleton algorithms were tested during our application development, and some were modified to match the situation better. It turned out that our algorithm has a good result both for correctness and calculation speed compared with the other algorithms. Our algorithm requires a shorter calculation time to find a maize plant’s skeleton, yields fewer unexpected branches and burrs, and the skeleton is located closer to the center axis. In Figure 13, we present our algorithm’s results compared with several other skeletonization algorithms. These candidate algorithms include Zhang’s thinning algorithm (Zhang and Suen, 1984), the media axis algorithm provided by scikit-image (van der Walt et al., 2014), and 2D skeleton extraction based on the heat equation (Gao et al., 2018). Figure 13A is the original segmentation image, and Figure 13B is the result of our skeleton algorithm. Figures 13C–E are the skeletonization results of Zhang’s thinning algorithm with branch pruning, the scikit-image’s media axis algorithm, and the heat equation 2D skeleton extraction, respectively.

The details of these skeleton images show that our method’s result has fewer unexpected branches, and the skeleton lies closer to the center axis. Although our method does not have the best result among these four methods, it has the highest efficiency. Supplementary Table 4 shows the calculation time consumption and memory use of our method and other methods. Our method is the fastest among these algorithms but consumes the largest amount of memory. With the development of smartphone chips, the processing memory of smartphones has become significantly larger. Therefore, our approach is a better choice for the skeletonization process on the smartphone platform.

### Efficiency of the Image Process Procedure

In general, the average execution time for a single plant image of 20 million pixels is 2,482 ms operating on an Android smartphone (Meizu MX4, MediaTek6595,CPU A17 2.2 GHz × 4 + A7 1.7 GHz × 4,GPU PowerVR G6200) All image process-related algorithms were developed using C++ language combined with the OpenCV library and compiled into a Java library for Android. The image processing procedure contains three major parts: segmentation, skeletonization and trait calculation. The average computational times are 1,050, 641, and 791 ms for segmentation, skeletonization and trait calculation, respectively. The total computation time for the whole procedure varied from 700 to 4,000 ms depending on the complexity of the plant structure and cleanliness of the background. Moreover, a faster segmentation algorithm that can reduce the process time to less than 100 ms is provided in the application for clean backgrounds with stable environments.

### Individual Leaf Traits Extraction

**FIGURE 14:**
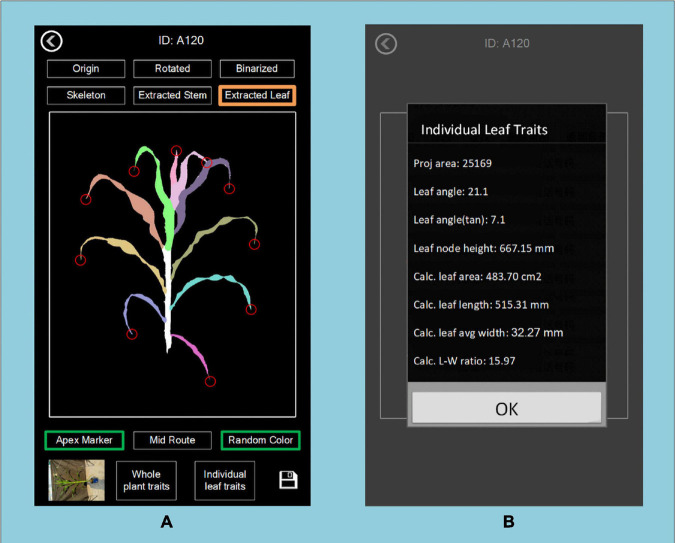
Display of individual leaf traits at different heights of the plant showing **(A)** the whole plant and **(B)** traits of a specific leaf.

PocketMaize provides an algorithm to extract all individual leaves from one maize plant and to obtain the traits of each leaf. It provides data for evaluating the difference between leaves at higher places and lower places. As shown in Figure 14, traits of individual leaves can be examined and stored for further analysis for canopy research and to investigate leaf overlap and sunlight absorption at different layers of the plant.

### Potential Application and Outlooks

Although the main object of this manuscript is to obtain single plant traits with high accuracy, the application can also calculate traits of several plants with minor overlapping, but the segmentation and traits calculation accuracy will decrease for severe overlapping. Moreover, the current work mainly focus on maize stem traits and leaf traits, the application to extract tassel traits and cob traits in reproductive stage will be improved in the future work. With training of enough images containing maize cobs and tassels, new segmentation model will be developed to obtain cobs traits during reproductive stage and estimate the final yield.

## Conclusion

In conclusion, we developed PocketMaize, an android smartphone application for maize plant phenotyping. The application is capable of field and potted maize phenotyping without many additional devices used. A total of 45 traits, which included 15 plant traits, 25 leaf traits and 5 stem traits, were nondestructively extracted. The average execution time for a single plant image of 20 million pixels was within 3,500 ms. In the future, with more trained images, a portable and cost-effective phenotyping solution could be extended to maize functional genomics studies, maize breeding, and disease and insect pest detection.

## Data Availability Statement

The raw data supporting the conclusions of this article will be made available by the authors, without undue reservation.

## Author Contributions

LL and WY designed the research, performed the experiments, analyzed the data, and wrote the manuscript. LY, DW, JY, and HF helped to perform the experiments. QL and WY supervised the project and helped to design the research. All authors contributed to the article and approved the submitted version.

## Conflict of Interest

The authors declare that the research was conducted in the absence of any commercial or financial relationships that could be construed as a potential conflict of interest.

## Publisher’s Note

All claims expressed in this article are solely those of the authors and do not necessarily represent those of their affiliated organizations, or those of the publisher, the editors and the reviewers. Any product that may be evaluated in this article, or claim that may be made by its manufacturer, is not guaranteed or endorsed by the publisher.
