# Supplementary material for: PocketMaize: An Android-Smartphone Application for Maize Plant Phenotyping
Source: Front Plant Sci. 2021 Nov 25;12:770217. doi: 10.3389/fpls.2021.770217 (PMC8656718; doi:10.3389/fpls.2021.770217)
Supplement: Supplementary file 1 [file Data_Sheet_1.docx]

**Supplementary Table 1** Stepwise regression analysis of total leaf area.

| **Model Summary^e^** | | | | | |
| --- | --- | --- | --- | --- | --- |
| Model | R | R Square | Adjusted R Square | Std. Error of the Estimate | Durbin-Watson |
| 1 | .820^a^ | .672 | .672 | 861.06628 |  |
| 2 | .839^b^ | .704 | .704 | 818.44284 |  |
| 3 | .862^c^ | .744 | .743 | 762.62168 |  |
| 4 | .871^d^ | .758 | .757 | 741.83065 | .776 |

| a. Predictors: (Constant), TLPA |
| --- |
| b. Predictors: (Constant), TLPA, TLL |
| c. Predictors: (Constant), TLPA, TLL, TLLxALW |
| d. Predictors: (Constant), TLPA, TLL, TLLxALW, MLW |
| e. Dependent Variable: TLA |

**Supplementary Table 2** Stepwise regression analysis of leaf dry weight.

| **Model Summary^f^** | | | | | |
| --- | --- | --- | --- | --- | --- |
| Model | R | R Square | Adjusted R Square | Std. Error of the Estimate | Durbin-Watson |
| 1 | .778^a^ | .605 | .604 | 515.94777 |  |
| 2 | .814^b^ | .663 | .662 | 476.98267 |  |
| 3 | .827^c^ | .684 | .682 | 462.33644 |  |
| 4 | .842^d^ | .710 | .708 | 443.09901 |  |
| 5 | .842^e^ | .710 | .709 | 442.85878 | .597 |

| a. Predictors: (Constant), TLPA |
| --- |
| b. Predictors: (Constant), TLPA, ALW |
| c. Predictors: (Constant), TLPA, ALW, TLL |
| d. Predictors: (Constant), TLPA, ALW, TLL, TLLxALW |
| e. Predictors: (Constant), TLPA, TLL, TLLxALW |
| f. Dependent Variable: LDW |

**Supplementary Table 3** Stepwise regression analysis of stem dry weight.

| **Model Summary^e^** | | | | | |
| --- | --- | --- | --- | --- | --- |
| Model | R | R Square | Adjusted R Square | Std. Error of the Estimate | Durbin-Watson |
| 1 | .910^a^ | .828 | .828 | 728.64460 |  |
| 2 | .916^b^ | .838 | .838 | 707.13796 |  |
| 3 | .920^c^ | .847 | .846 | 688.66994 |  |
| 4 | .922^d^ | .849 | .848 | 683.62784 | 1.510 |

| a. Predictors: (Constant), SPA |
| --- |
| b. Predictors: (Constant), SPA, SV |
| c. Predictors: (Constant), SPA, SV, SH |
| d. Predictors: (Constant), SPA, SV, SH, SW |
| e. Dependent Variable: SDW |

**Supplementary Table 4** A comparison of four skeletonization methods ^a^

| Methods of skeletonization | Process time/ms | Burr | Memory used/MB |
| --- | --- | --- | --- |
| Our method | 738 | ++ | 595 |
| Zhang’s thinning algorithm with branch pruning | 1570 | +++ | 70 |
| Scikit-image’s skeletonization | 48770 | +++ | 95 |
| Heat equation based skeletonization | 1372 | + | 155 |

a. The average data for 300 testing images
